# Zinc Prevents Abdominal Aortic Aneurysm Formation by Induction of A20-Mediated Suppression of NF-κB Pathway

**DOI:** 10.1371/journal.pone.0148536

**Published:** 2016-02-26

**Authors:** Ya-Wei Yan, Jun Fan, Shu-Ling Bai, Wei-Jian Hou, Xiang Li, Hao Tong

**Affiliations:** 1 Department of Tissue Engineering, School of Fundamental Science, China Medical University, Shenyang, China; 2 Department of Cell Biology, College of Basic Medicine, China Medical University, Shenyang, China; University of Illinois at Chicago, UNITED STATES

## Abstract

Chronic inflammation and degradation of elastin are the main processes in the development of abdominal aortic aneurysm (AAA). Recent studies show that zinc has an anti-inflammatory effect. Based on these, zinc may render effective therapy for the treatment of the AAA. Currently, we want to investigate the effects of zinc on AAA progression and its related molecular mechanism. Rat AAA models were induced by periaortic application of CaCl_2_. AAA rats were treated by daily intraperitoneal injection of ZnSO_4_ or vehicle alone. The aorta segments were collected at 4 weeks after surgery. The primary rat aortic vascular smooth muscle cells (VSMCs) were stimulated with TNF-α alone or with ZnSO_4_ for 3 weeks. The results showed that zinc supplementation significantly suppressed the CaCl_2_-induced expansion of the abdominal aortic diameter, as well as a preservation of medial elastin fibers in the aortas. Zinc supplementation also obviously attenuated infiltration of the macrophages and lymphocytes in the aortas. In addition, zinc reduced MMP-2 and MMP-9 production in the aortas. Most importantly, zinc treatment significantly induced A20 expression, along with inhibition of the NF-κB canonical signaling pathway in vitro in VSMCs and in vivo in rat AAA. This study demonstrated, for the first time, that zinc supplementation could prevent the development of rat experimental AAA by induction of A20-mediated inhibition of the NF-κB canonical signaling pathway.

## Introduction

Abdominal aortic aneurysm (AAA) is a kind of serious vascular disease with high incidence and high mortality. Furthermore, with the change of the lifestyle and an aging population, the incidence is a rising trend [1]. Its typical pathological changes include chronic inflammatory cells infiltration, aortic elastin proteolytic degradation and pathological remodeling. These changes result in the destruction of elastic lamellar structure in the aortic media and therefore gradual aneurysmal dilatation and even finally rupture [2].

Inflammation plays significant role in the progression of AAA [3,4], which may be the potential treatment target for AAA. Recent studies have shown that zinc finger protein A20 could prevent inflammatory response in aortic allografts and development of transplant arteriosclerosis [5]. Zinc finger protein A20, a zinc-finger transactivating factor, was identified as a primary response gene following inflammatory stimulation (TNF, IL-1 or LPS) of human umbilical vein endothelial cells [6]. A20 can also be induced in smooth muscle cells and exhibit an anti-inflammatory impact by blockade of nuclear factor κB (NF-κB) signaling [7, 8]. NF-κB can promote chronic inflammation in the aortic wall [9], and regulate MMPs transcription [10]. In human and animals experiment, inhibition of NF-κB activation can prevent the development of AAA [11, 12].

Zinc is one of the most common trace elements in the human body, and needed for DNA synthesis, RNA transcription, cell division and activation. Zinc plays a critical role in wound healing, biosynthesis, and homeostasis of various connective tissues [13]. Zinc also has anti-inflammatory action obviously regulating pathogenesis of the inflammation-related diseases [14]. Reports have shown that decreased plasma zinc and increased inflammatory cytokines in the elderly subjects were corrected by zinc supplementation [15]. At same time, zinc deficiency will induce vascular inflammation associated with NF-κB signaling [16]. Zinc can induce A20 expression and inhibit NF-κB activation, decrease incidence of infection and generation of inflammatory cytokines in patients [17]. Based on these, we speculate that zinc may render effective therapy for the treatment of the AAA.

In the present study, the purpose is to detect whether zinc supplementation can prevent the development of experimental AAA, with special concentration on the regulation of A20-NF-κB pathway in vivo and vitro studies.

## Materials and Methods

### Experiment agents and instruments

Anti-A20, anti-elastin antibody was obtained from Santa Cruz Technology (Santa Cruz Biotech, Santa Cruz, CA, USA). Primary polyclonal antibodies against phospho-IKKβ (Ser180/181), phospho-IκBα (Ser32/36), IKKβ, IκBα, and NF-κB p65 are from Cell Signaling Technology. Anti-CD45, anti-CD68 and anti-CD20 were from Boster, China. Anti-β-actin, anti-MMP-2, anti-MMP-9 were purchased from Bioss, China. Calcium chloride anhydrous (CaCl_2_) and zinc sulfate heptahydrate (ZnSO_4_·7H_2_O) were purchased from Sigma. Diaminobenzidine (DAB) and strept-avidin biotin complex (SABC) immunohistochemical kit were purchased from Boster (Wuhan, China). Fetal bovine serum and Dulbecco's modified Eagle's medium (DMEM) were purchased from Hyclone (Logan, Utah, USA).

### Animal experiments

30 adult male 8-wk-old Wistar rats (obtained from the experimental animal center of China Medical University) weighing 250 to 300 g were randomly divided into three groups: control group (without any treatment), AAA group (treated by CaCl_2_ alone), zinc-administrated group (treated by CaCl_2_ together with zinc intraperitoneal injection), with 10 rats per group. All animal experiments were carried out in strict accordance with the recommendations in the Guide for the Care and Use of Laboratory Animals of the National Institutes of Health. The protocol was approved by the Institutional Animal Care and Use Committee (IACUC) of China Medical University. The approval reference number is SCXK (Liao) 2013–0001. All rats were housed under a 12-hour light/dark cycles and had free access to a normal diet and water ad libitum. Rat AAA was established by perivascular application of 0.5 M CaCl_2_ as previously described [18], whereas saline was used in the control group. In short, the rats were anesthetized with sodium pentobarbital (40mg/kg, intraperitoneally), sheared and disinfected, then fixed on the operating table in the supine position. An incision was made on the abdominal median to expose the abdominal aorta. Then sponge containing CaCl_2_ was directly placed on the abdominal aorta at approximately the center of the section between the renal artery and the iliac bifurcation. The sponge was removed after 15 min followed by closing the abdominal cavity in layers.

After the surgery, zinc-administrated group animals were immediately provided with daily intraperitoneal injection of ZnSO_4_ at a dose of 3 mg/kg/day (physiological dose) [19, 20] for 4 weeks. AAA group rats were injected with the equivoluminal saline.

Four weeks after the operation, these rats were anesthetized with sodium pentobarbital (60mg/kg, absolute lethal dose, intraperitoneally) and treated aortic segments were excised, fixed in 4% phosphate-buffered paraformaldehyde, embedded in paraffin for the following assays. Changes of the aortic external diameter were analyzed by applicating SZH stereomicroscope.

### Histological examination (Orcein staining, HE staining)

Serial sections of the abdominal aortic segments with Orcein staining and HE staining according to the standard techniques were used to observe the changes of the elastic lamellae and the morphology of aortic walls, respectively. The experimental results were analyzed by using MetaMorph/DP10/BX41 microscope image analysis system.

### Immunohistochemical staining

Anti-CD45 (1:200, leucocytes, Boisynthesis, China), anti-CD68 (1:100, macrophages, Boster Biotechnology, China) and anti-CD20 (1:100, B lymphocytes, Boster Biotechnology, China) were used to locate and specify inflammatory cells infiltration in the abdominal aortic walls. Immunohistochemical staining was performed on 5μm sections. They were dewaxed, rehydrated in graded alcohols, blocked for inhibiting endogenous peroxidase activity by using 3% hydrogen peroxidase, and preincubated with 5% normal bovine serum albumin (BSA). Appropriate diluted rabbit polyclonal primary antibodies were applied to the sections. Subsequently, they were serially incubated with biotinylated anti-mouse IgG and the supersensitive strept avidin-biotin complex (SABC) according to the manufacturer’s directions. Immune complexes were visualized by using 3, 3'-diaminobenzidine (DAB), and cell nucleus were counterstained with hematoxylin. All sections were dehydrated and covered by cover glasses with neutral balsam. For the negative control experiments, we substituted phosphate buffer solution (PBS) for the primary antibody. The infiltration of inflammatory cells was determined by counting the mean number of nuclei surrounded by positive immunostaining within 400× high power fields (HPF). Non-specific staining was not observed.

### Cell culture and experiments

Primary aortic VSMCs were obtained by explant cultures as previous method [21]. Adult healthy Wistar rats were anesthetized with sodium pentobarbital (60mg/kg, absolute lethal dose, intraperitoneal) and then their aortas were excised from the section between the renal artery and the iliac bifurcation, separated loose connective tissue and collateral vessels, scraped off intimal layer, then cut into 1–2 mm^2^ small segments and transferred into 25 cm^2^ tissue culture flasks supplemented with high glucose DMEM (H-DMEM) plus 20% foetal bovine serum (FBS), 100 U/mL penicillin, and 100 U/mL streptomycin, After 4 days, cells freed out around the tissue and subculture was done when cells reached 80% confluence. After the third passage, the VSMCs were identified with Immunofluorescence assay for α-SMA (α-smooth muscle actin).

The fourth to sixth passage VSMCs were treated with no-additives, TNF-α alone and TNF-α together with zinc. TNF-α and zinc sulfate heptahydrate were dissolved in DMEM and added at 10ng/mL and 15μM respectively, then cultured continuously for 3 weeks, medium was replaced every 3 days. The all cells were serum-starved in DMEM containing no FBS for 24 h to synchronize them in G_0_ phase of the cell cycle prior to treatments.

### Western Blotting

Total proteins were extracted from the abdominal aortic tissues and variously treated VSMCs. The protein concentrations were determined using a BCA protein assay kit (Beyotime). After denaturing the proteins with loading buffer, the same amount of protein (40 mg)and 2 μl pre-stained protein molecular weight marker were used for Western blot analysis with primary antibodies against A20 (1:500, Santa Cruz), phospho-IKKβ(1:800, Ser180/181; Cell Signaling), phospho-IκBα (1:800, Ser32/36;Cell Signaling), IκBα (1:1000, Cell Signaling), and NF-κB p65 (1:1000, Cell Signaling), MMP-2, 9 (1:300, Boster, China) with β-actin(1:1000, Bioss, China) as positive control, and with the respectively appropriate HRP-conjugated secondary antibody. Specific antibody binding was subsequently visualized using an enhanced chemiluminescence (ECL) kit (Beyotime Biotechnology, Shanghai, China) following the manufacturer’s instructions using ChemDoc XRS with Quantity Onesoftware (BioRad, Hercules, CA). The experiments were repeated three times. The intensity of each band determined by densitometry using image analyzing software indicated the expression level of the specific-protein.

### Statistical analysis

All values were expressed as the mean±SEM. Analysis of variance was used to determine the significance of differences in multiple comparisons. These results were processed using the Statistical Package for the Social Sciences, version 16.0 (SPSS Inc., Chicago, IL, USA). Comparisons between groups were conducted using the T test. P <0.05 was considered statistically significant.

## Results

### Zinc supplementation prevented the progression of CaCl_2_-induced AAAs

**Fig 1:**
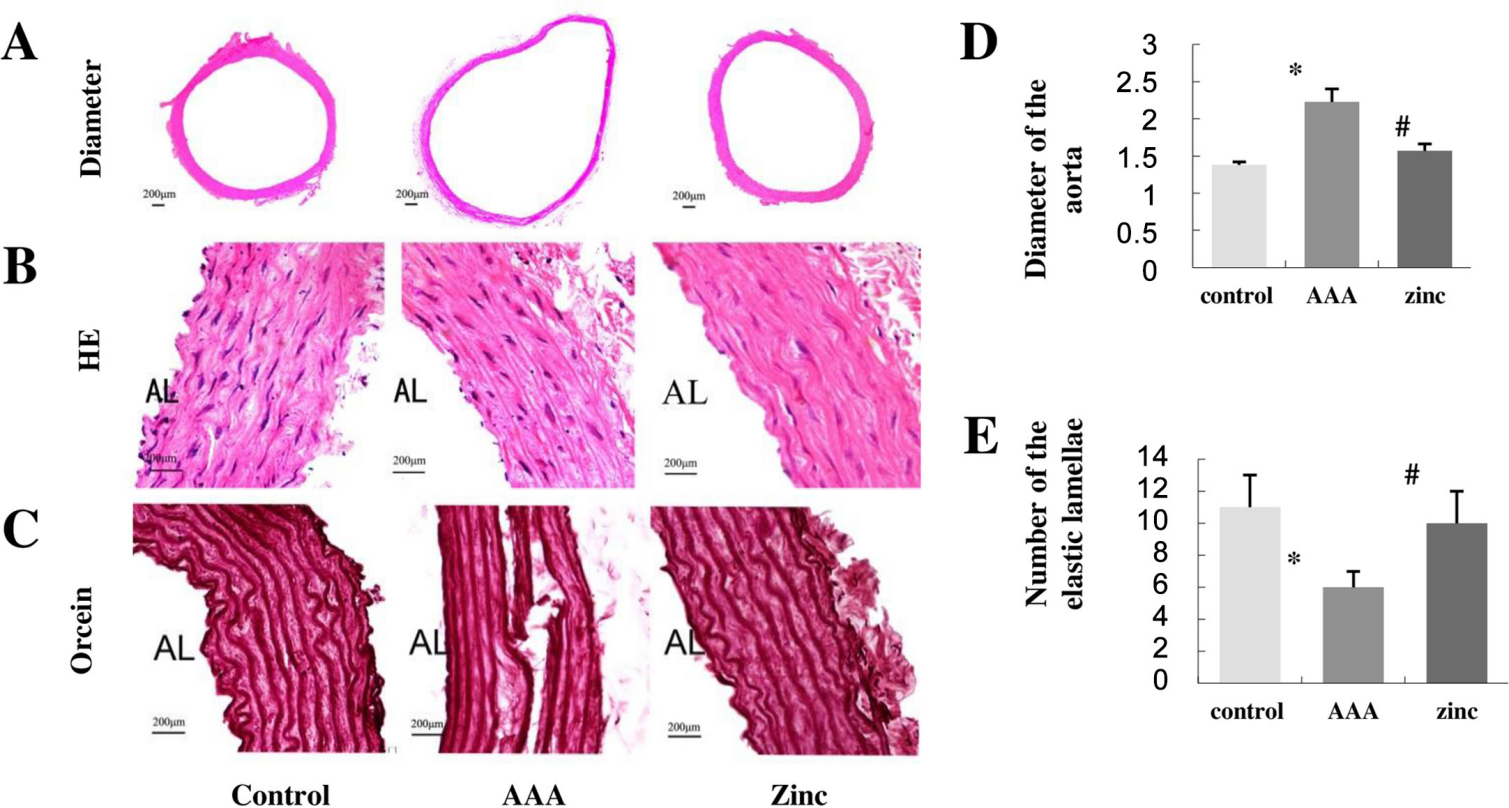
Effect of zinc on the AAA. (A) Representative images of the diameters of the aortas. (B) Representative macroscopic morphological changes by low-power micrographs of hematoxylin and eosin staining. (C) Representative orcein staining showing elastic lamellae changes of AAA sample. (D) The diameter of the aortas. (E)The number of the elastic lamellae. AL: aortic lumen. Scale bars: 200μm. Results were presented as mean±SEM. n = 10 for each group. * p < 0.05 versus control group, # p < 0.05 versus AAA group.

Four weeks after CaCl_2_-induced AAA surgery, the rats were sacrificed and the aorta tissues were collected. First, the inhibitory effects of zinc on the AAA development were determined by observing the aneurysmal incidence, aortic external diameter and the morphological changes. The aneurysmal incidence in the AAA group was 70%, while that in the zinc-administrated group was only 20%. The aortic external diameter in the AAA group increased obviously compared with that in the control group (control group, 1.38±0.02mm; AAA group, 2.43±0.17mm). However, the administration of zinc significantly decreased the aortic diameter compared with the AAA group (1.57±0.09 mm) (Fig 1A and 1D).

HE staining showed that there were orderly and clear layers in the abdominal aortic walls in the control group, while the abdominal aortic walls in the AAA group became disorganized and thinner. However, the abdominal aortic walls in the zinc-administrated group had almost the same morphology as the normal abdominal aorta (Fig 1B).

Aortic extracellular matrix proteolytic degradation is one of the main characteristic changes of the aneurysm. Orcein staining was used to detect the elastin changes in the aorta. Orcein staining showed that the elastic lamellae of the control group were corrugated appearance and integrated structure without any damage. The elastic lamellae in the AAA group were flat or even disrupted and dispersed. But zinc-supplementation preserved the wave elastic lamellae and alleviated the elastin fragmentation (Fig 1C).

In addition, zinc-supplementation reversed the decreased elastic lamellae number in the wall of AAA. Orcein staining demonstrated that the elastic lamellae number was 11±2 in the control group, 6±1 in the AAA group and 9±2 in the zinc-administrated group (Fig 1E). At same time, the interval of elastic lamellae increased in the AAA group compared with that in the control group, while tight elastic lamellae are similar to the normal aortic wall in the zinc-administrated group.

### Zinc preserved the elastin

**Fig 2:**
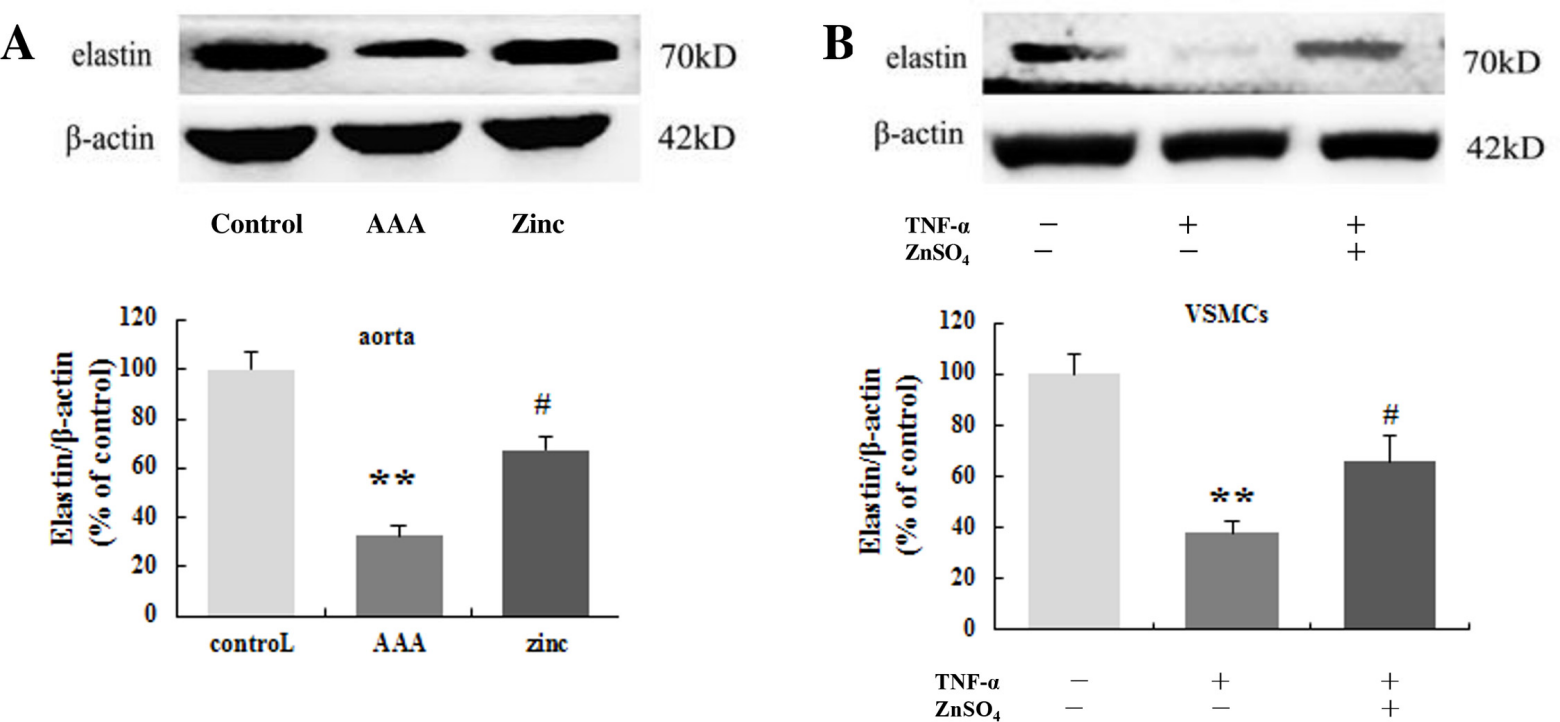
Zinc preserved the elastin in the VSMCs in vitro. (A, n = 3 independent experiments) and in the aortas in vivo (B, n = 10 for each group). The β-actin was used as an internal control. Results were presented as mean±SEM. **p < 0.01versus control group, # p < 0.05 versus AAA group (or TNF-α group).

Primary aortic VSMCs were cultured. At 3 day of culture, some cells appeared around the tissue aorta segments. The cells exhibited typical spindle-like morphology under optical microscope (S1 Fig). In addition, the cells were stained with α-SMA antibody, no non-staining cells were observed (S1 Fig). The results confirmed that the isolated cells were VSMCs. VSMCs were treated with TNF-α to mimic the inflammation environment of the AAA in vivo. Western blot exhibited TNF-α treatment decreased the elastin level in the VSMCs, while the VSMCs treated with TNF-α together with zinc showed significantly higher expression level of elastin than that in treated with TNF-α alone (Fig 2A). Western blot further exhibited that there was more elastin expression in the aorta segments of the zinc-administrated group than that of the AAA group (Fig 2B). Therefore we concluded that zinc could prevent the elastin degradation in VSMCs in vitro and in AAA in vivo.

### Zinc reduced the inflammation in the AAA

**Fig 3:**
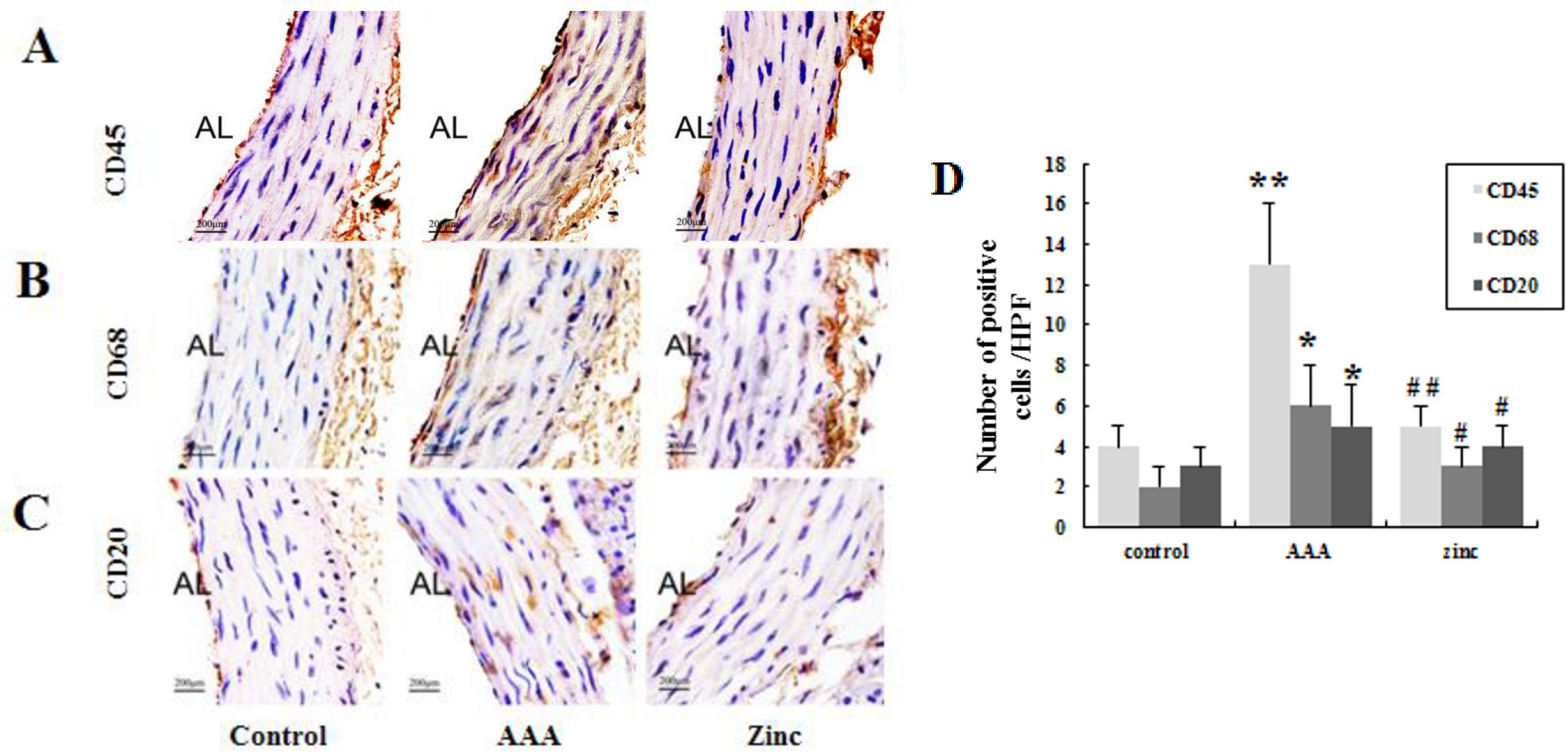
Immunohistochemical staining showed the infiltration of the inflammatory cells. CD45 (A), CD68 (B) or CD20 (C) positive cells in vessel walls. (D), the number of the inflammatory cells by statistical analysis. Brown deposits indicate positive staining. Results were presented as mean±SEM. n = 10 for each group. * p < 0.05 versus control group, # p < 0.05 versus AAA group. AL: aortic lumen. Scale bars: 200μm.

To evaluate the effect of zinc on the inflammation in AAA, the immunostaining of leukocytes (CD45), macrophages (CD68) and B cells (CD20) in aortas was conducted. The number of CD45+, CD68+ and CD20+ cells was increased in aortas of AAA group versus control group, indicating that infiltration of macrophages and B cells were increased in AAA. However, zinc-administration abolished leukocytes, macrophages and B cells infiltration in aortas of AAA group, suggesting that zinc can reduce the inflammation in the AAA as shown in Fig 3.

### Zinc induced the expression of A20 in abdominal aorta in vivo and VSMCs in vitro

**Fig 4:**
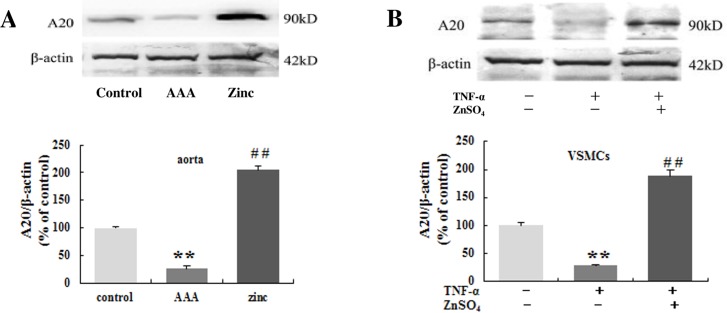
Zinc induced the expression of the A20 in the aortas in vivo (A, n = 10 for each group) and in the VSMCs in vitro (B, n = 3 independent experiments). The β-actin was used as an internal control. Results were presented as mean±SEM. ** p < 0.01versus control group, ## p < 0.01 versus AAA group (or TNF-α group).

Previous studies showed that zinc can induce A20 protein expression by inhibiting inflammation [22, 23]. We examined expression of A20 protein in abdominal aorta segments and primary cultures of VSMCs treated as described in Material and methods. In vivo experiment, there was lower A20 expression in the AAA group aortas than that in the control group aortas. However, zinc supplementation significantly reversed the low A20 expression in abdominal aortas (Fig 4A). In vitro experiment, the primary VSMCs were treated with TNF-α in the presence or absence of zinc, TNF-α alone decreased A20 expression, but combined with zinc could increase A20 expression (Fig 4B).These results demonstrated that A20 expression can be induced by zinc administration in AAA.

### Zinc inhibited the NF-κB activation

**Fig 5:**
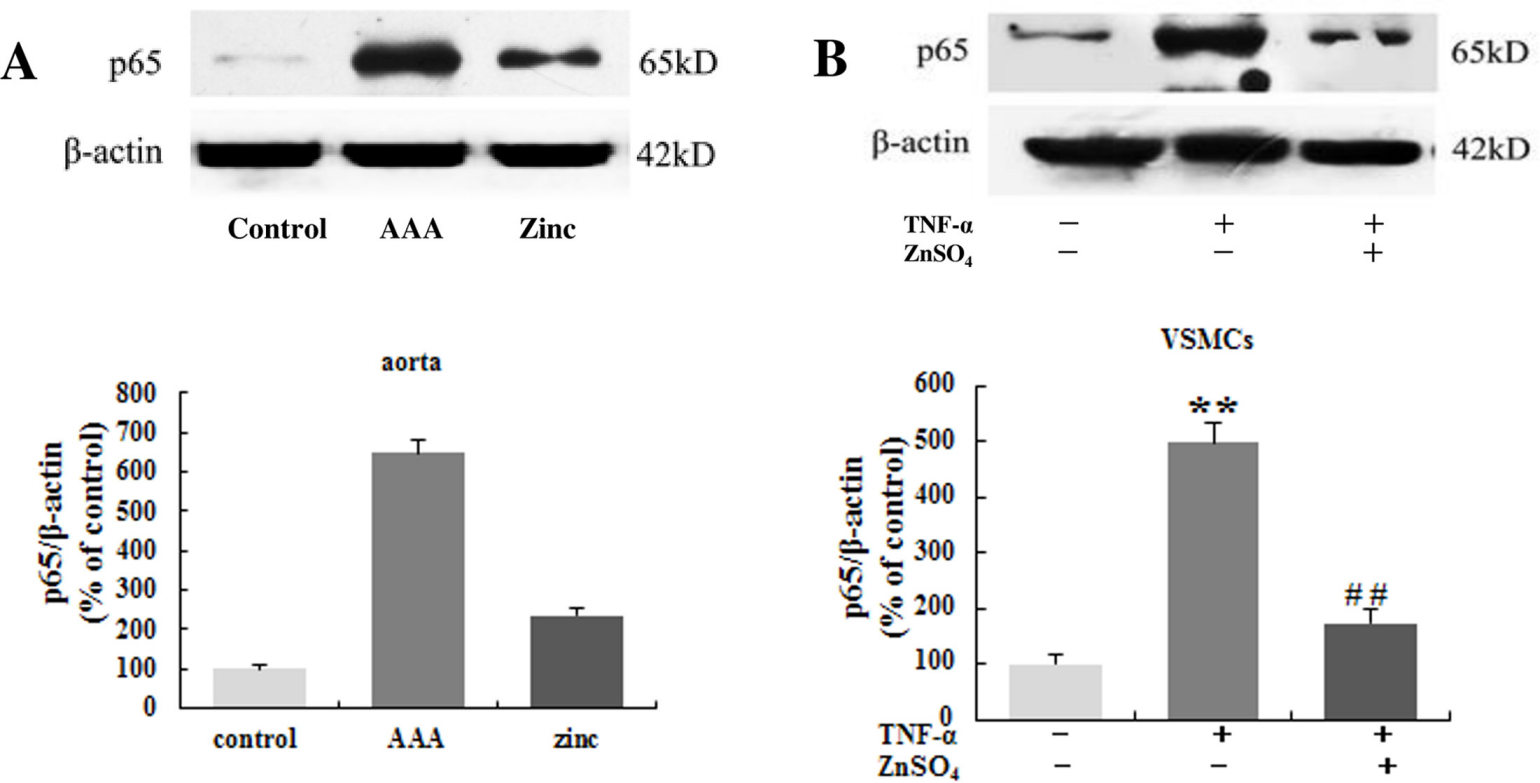
Zinc inhibited the activation of the NF-κB in the aortas in vivo (A, n = 10 for each group) and in the VSMCs in vitro (B, n = 3 independent experiments). The β-actin was used as an internal control. Results were presented as mean±SEM. ** p < 0.01versus control group, # #p < 0.01 versus AAA group (or TNF-α group).

NF-κB activation is pivotal to the inflammation process. Therefore, we want to know whether zinc can inhibit the NF-κB activation in the AAA. The NF-κBp65 subunit only slightly expressed in the aortas in the control group, but its expression evidently increased in the AAA group. However, zinc-administration apparently reduced NF-κBp65 expression in the aortas (Fig 5A). In vitro experiment, TNF-α alone obviously increased the NF-κBp65 subunit expression in the VSMCs compared with control group, while NF-κBp65 subunit expression was significantly down-regulated in the VSMCs in TNF-α together with zinc group(Fig 5B).The above results indicated zinc inhibited the NF-κB activation in the AAA.

### Zinc decreased the phospho-IKKβ, phospho-IκBα and prevented the degradation of IκBα

**Fig 6:**
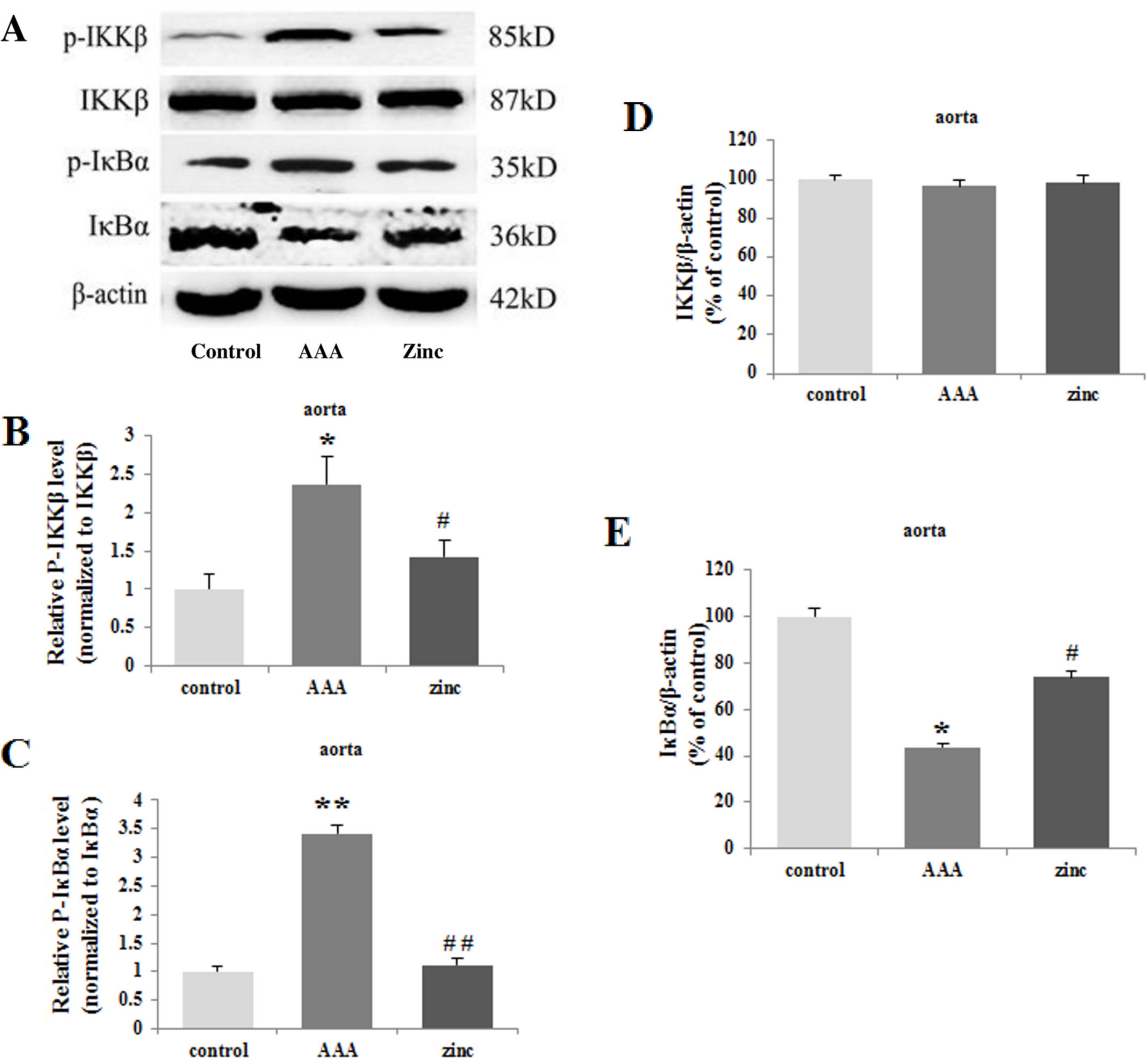
**The expression of the p-IKKβ, p-IκBα, IKKβ and IκBα in the abdominal aortas in vivo (A). Zinc decreased the phospho-IKKβ (B), phospho-IκBα (C) and degradation of IκBα (E).** The level of the IKKβ had no significant difference (D).The β-actin was used as an internal control. Results were presented as mean±SEM. n = 3 independent experiments.* p < 0.05 versus control group, # p < 0.05 versus AAA group.

**Fig 7:**
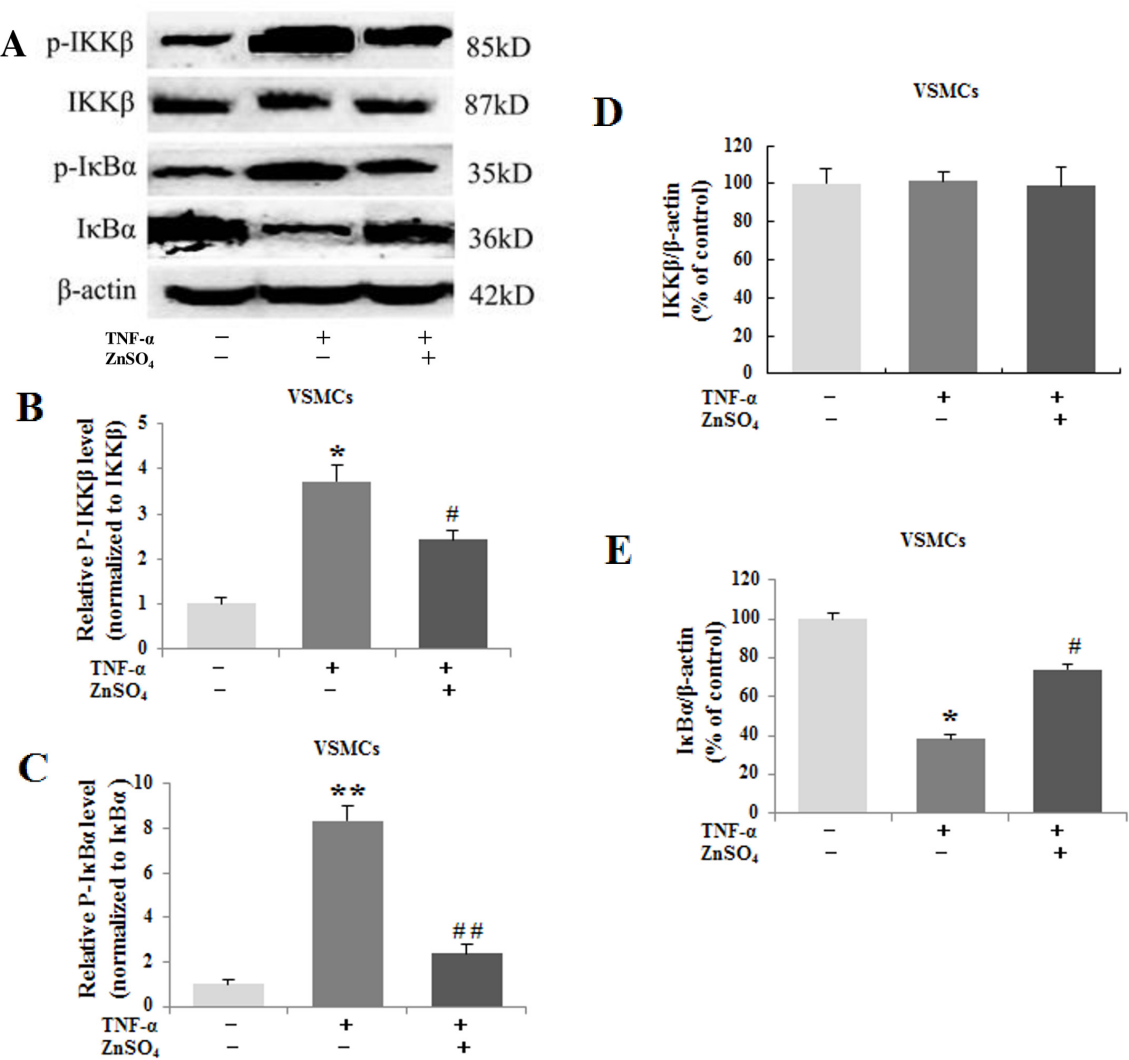
**The expression of the p-IKKβ, p-IκBα, IKKβ and IκBα in the VSMCs in vivo (A). Zinc decreased the phospho-IKKβ (B), phospho-IκBα (C) and degradation of IκBα (E).** The level of the IKKβ had no significant difference (D).The β-actin was used as an internal control. Results were presented as mean±SEM. n = 3 independent experiments.*p < 0.05 versus control group, # p < 0.05 versus AAA group.

The canonical NF-κB signaling regulates expression of large number of molecules involved in inflammation. The effect of zinc on the canonical NF-κB signaling was detected. Compared to the control group, the p-IKKβ and p-IκBα increased significantly, while the IκBα decreased distinctly in the abdominal aortic walls in the AAA group. However, zinc administration remarkably reduced the p-IKKβ and p-IκBα and prevented the IκBα degradation (Fig 6).In vitro experiment, TNF-α alone obviously increased the generation of p-IKKβ and p-IκBα and degraded the IκBα in the VSMCs compared with that in the control group. But, the generation of p-IKKβ and p-IκBα clearly decreased and the IκBα significantly increased in the VSMCs in the presence of zinc (Fig 7). IKKβ level was similar among different groups in vivo and in vitro.

### Zinc alleviated the MMP-2 and MMP-9 expression in the AAA

**Fig 8:**
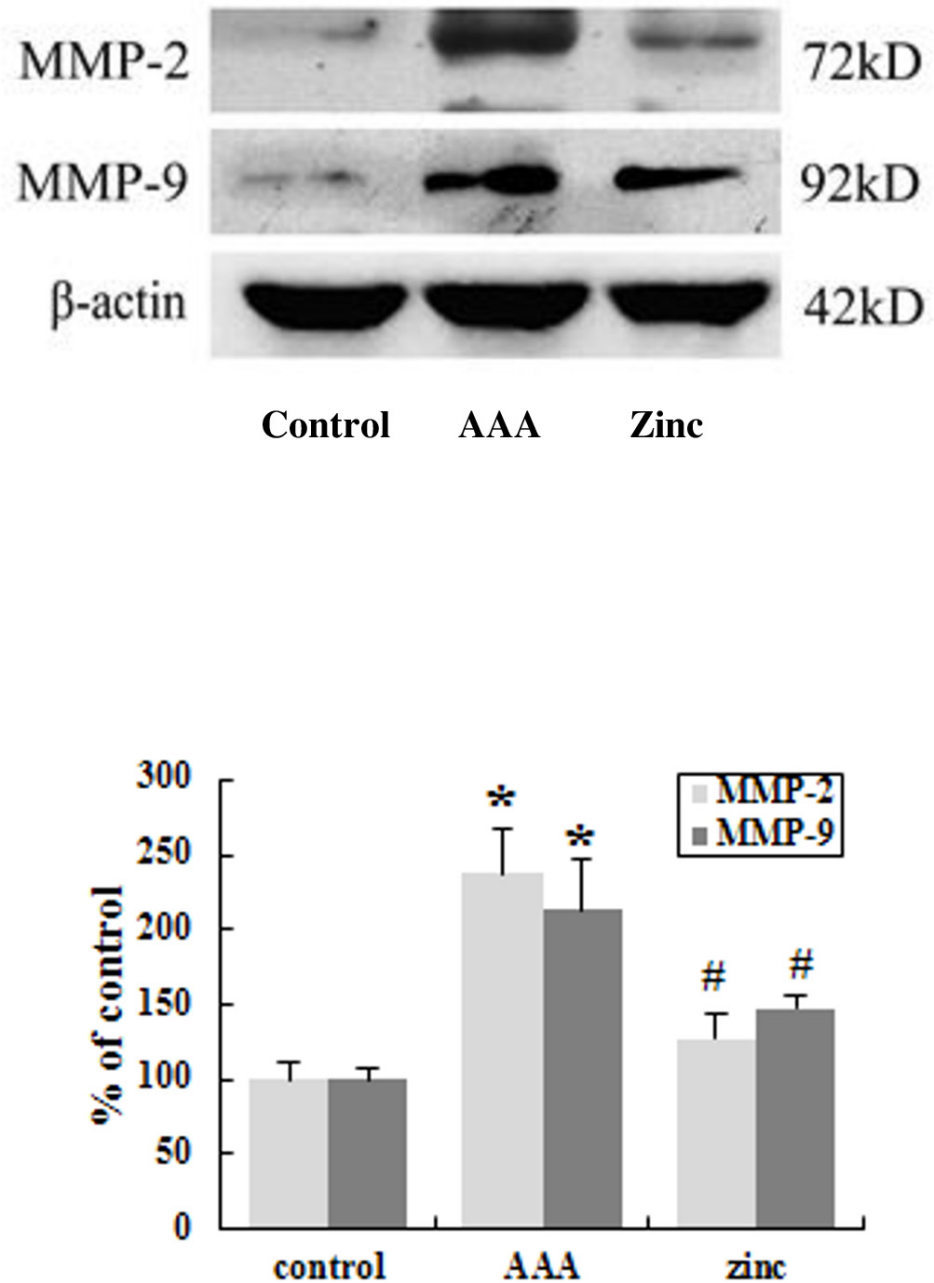
Zinc reduced the expression of the MMP-2 and MMP-9 in abdominal aortas in vivo. The β-actin was used as an internal control. Results were presented as mean±SEM. n = 10 for each group.* p < 0.05 versus control group, # p < 0.05 versus AAA group.

MMP-2 and MMP-9 could degrade elastic fibers and were considered as especially important molecules in the pathogenesis of AAAs [24]. Western blot result revealed that the expression of the MMP-2 and MMP-9 was significantly up-regulated in the aortas of the AAA group compared with that of the control group, while zinc-administration significantly decreased the MMP-2 and MMP-9 expression in the aortas (Fig 8). So we confirm that zinc supplementation can inhibit the expression of the MMP-2 and MMP-9 in the AAA.

## Discussion

Our results demonstrated that zinc supplementation significantly suppressed the abdominal aorta expansion, preserved elastin fibers and prevented the development of experimental AAA. Furthermore, studies revealed that zinc could reduce the infiltration of inflammatory cells and the expression of MMP-2 and MMP-9 in AAA. We also found that zinc induced A20 expression and inhibited the canonical NF-κB signaling pathway in vitro in VSMCs and in vivo in AAA. These findings point to a new strategy to treat AAA.

Inflammation is one of the main reasons responsible for the development of AAA. The inflammatory cells produce MMPs to degrade the elastin in the aortic wall, especially MMP-2 and MMP-9. In our experiment, the AAA in rats was induced by periaortic application of calcium chloride (CaCl_2_) [25]. The inflammatory aspect of our animal AAA resembles that found in human AAAs. TNF-α which has a pivotal role in the process of inflammation [23] is a particularly dominant cytokine secreted by inflammatory cells within aneurysms. TNF-α mediates inflammatory response and incites MMPs release, down-regulate elastin gene expression and initiates aneurysms [26, 27]. In the present experiment, VSMCs as the main elements of the aorta were treated with TNF-α for 3 weeks to resemble the AAA in vivo. The concentration and the time of TNF-α were chosen based on previous reports which suggest the culture system closely mimic chronic, long-term TNF-α-induced signaling in aneurysms [28].

Zinc plays an important role in cell-mediated immune functions and also works as an antioxidant and anti-inflammatory agent [12, 29]. Previous observation revealed decreased plasma zinc, increased plasma oxidative stress markers and increased generation of inflammatory cytokines in the elderly subjects which were corrected by zinc supplementation [28]. In the AAA patients group, the zinc levels in serum were significantly lower than those of the control group. So we hypothesized that zinc supplementation might down-regulate the inflammation and prevent the development and progression of AAA.

Elastin is one of the major structural proteins of aortic wall and plays an indispensable role in maintaining the extensibility and elastic recoil of the aortas [30]. As an essential trace element, zinc plays a critical role in biosynthesis and homeostasis of various connective tissues. Peter Takacs and Mahoney et al have shown zinc could increase tropoelastin production in vaginal smooth muscle cells and human skin cells [13, 31]. Zinc is also involved in cell proliferation and DNA synthesis. Zinc deficiency decreases gene expression of DNA-synthesizing enzyme deoxy thymidine kinase (TK) in HUT 78 cells [32]. Additionally, in human study, zinc deficiency reduces the activity of deoxy TK in the implanted sponge connective tissue [33]. We hypothesized that zinc supplementation might prevent AAA development process by inducing elastin composition. In the current study, zinc administration significantly decreased the abdominal aortic external diameters. The aneurysmal incidence in the AAA group was 70%. But the incidence of AAA was only 20% in the zinc-administrated group. Additionally, the AAA group aortas presented severely disorganized wall structure and remarkably reduced cells in thin media. However, the zinc-administrated group aortas had almost the same morphology as the control group aortas. The Orcein staining showed that the elastic lamellae in the AAA group became flatten or even disrupted and dispersed, but zinc-supplementation preserved the wave elastic lamellae and alleviated the elastin fragmentation in the aorta. Western blot also exhibited that the elastin expression in the abdominal aortic segments of the zinc-administrated group increased compared with that of the AAA group. Since VSMCs are the main component of the aorta and involved in the destruction and biosynthesis of the elastin, the elastin level was detected in the VSMCs in vitro treated with TNF-α alone or with zinc. Primary VSMCs treated with TNF-α together with zinc expressed significantly higher level of elastin than that treated with TNF-α alone. From above results, we concluded that zinc could preserve the elastin in aortic segments in vivo and VSMCs in vitro.

Chronic inflammation played a pivotal role in the destruction of elastic lamellae in the pathogenesis of AAA [13]. In the aneurysmal wall, most of the cells within the inflammatory infiltrates were positive for the leukocyte common antigen (CD45), macrophages (CD68) and B-lymphocytes (CD20), only scarce T-lymphocytes and neutrophil leukocytes [34]. The inflammatory cells are the major source of MMPs. MMP-2 and MMP-9 are predominant responsible for proteolytic degradation of elastin which plays an important role in the development of AAA [4]. The increased aortic diameters are associated with inflammatory infiltration and MMP-2 and MMP-9 secretion. Treatment with anti-inflammatory agents or MMPs antagonists can lead to preservation of elastin in the media and reduction in experimental aneurysm development [4]. Previous reports suggested that zinc not only plays a crucial role in biosynthesis and homeostasis of various connective tissues but also has anti-inflammatory function regulating pathogenesis of the inflammation-related diseases [16, 25]. Our results demonstrated that in the AAA group, macrophages and B lymphocytes existed in all layers of the abdominal aortic wall, which was consistent with the previous reports [3]. However, the zinc treatment significantly decreased inflammatory cells infiltration in AAA. At same time, zinc treatment significantly decreased the expression of MMP-2 and MMP-9 in the AAA. Based on these results, we confirmed that zinc treatment could suppress the inflammation and the expression of MMP-2 and MMP-9 in abdominal aortic aneurysm.

Zinc finger protein A20 was initially identified as a primary response gene following stimulation of human umbilical vein endothelial cells (HUVEC) with TNF, IL-1 or LPS [6]. Subsequently, A20 was also found to be transcriptionally upregulated in many other cell types by a wide range of other stimuli. A20 is a potent anti-inflammatory protein and plays critical roles in the process of inflammation. A20 can prevent inflammatory response in aortic allografts and development of transplant arteriosclerosis [35]. Zinc supplementation may lead to downregulation of the inflammatory cytokines by upregulation of the negative feedback loop A20 inhibiting NF-κB activation [36]. In the current experiment, the expression of A20 was examined in abdominal aorta segments and primary VSMCs treated following the way mentioned in material and methods. There was lower A20 expression in the aortas in AAA group compared with that in the control group. However, zinc supplementation significantly reversed the low A20 expression in abdominal aortas. In VSMCs, TNF-α alone decreased A20 expression, but combined with zinc could increase A20 expression. Recent study demonstrated A20 gene knockout mice die shortly after birth by severe inflammation and tissue damage in multiple organs [37]. But A20 overexpression substantially inhibited NF-κB activation and decreased expression of several NF-κB target genes, such as E-selectin, ICAM-1, IL-8 and IκBα [38]. Moreover, A20 can terminate canonical NF-κB activation by deubiquitinating key signaling molecules in the TNFR, IL-1R, and TLR signaling pathways, and inhibit the expression of TNF-α, IL-1β and proteins generated through the NF-κB-inducible kinase and Iκ kinase (IKK)-β-NF-κB pathways [39]. But some studies were not consistent with above experiments, which exhibited that TNF-α injection and injury in mice resulted in a substantial up-regulation of A20 in many organs. The reasons may lie in that A20 induced by inflammatory stimuli is transient and NF-κB activation inhibited by A20 is cell specific. The concreted mechanism need to be further investigated. The NF-κB family counts five subunits: RelA or p65, RelB, c-Rel or Rel, p50 and p52, and plays a key role in several cellular functions, including inflammation, apoptosis, cell survival, proliferation, angiogenesis, innate and acquired immunity, and so on [40]. The NF-κB includes two independent, yet interlinked, signaling pathways: the canonical or classical pathway and the non-canonical or alternative pathway. In vivo and in vitro studies have clarified that the two NF-κB pathways have several different functions which depend on the cell types and the stimulus applied. As the main NF-κB signaling pathway in many cell types, the canonical pathway regulates the expression of inflammatory response molecules and MMPs, and suppresses the transcription and synthesis of elastin [41]. The activation of the NF-κB is the precondition of the inflammatory response and is considered to be one of the key factors in development and progression of AAA [42, 43]. Recent studies have shown that NF-κB p65 can promote inflammatory changes and regulate MMPs (MMP-1, MMP-2, MMP-3, and MMP-9) transcription participating in the initiation and progression of AAA and intracranial aneurysm (IA) [44]. In IAs, the activation of the NF-κB and the expression of MMP-2 were positive correlation. Inhibition of the NF-κB can reduce the expression of MMP-9 in fibroblasts and VSMCs. Also, inhibition of NF-κB or MMP-2 and MMP-9 can effectively prevent the development of AAA. Chimeric decoy oligonucleotides can prevent AAA in a rabbit model through inhibiting the NF-κB [12]. Zinc has been depicted as both a negative and positive regulator of NF-κB, its effect on the canonical NF-κB pathway may be apparently different in diverse cell types and under various zinc concentrations [45]. Therefore, the expression levels of p65, p-IKKβ, p-IκBα and IκBα were detected under the conditions in the abdominal aortas and in VSMCs. In vivo experiment, compared with the control group, the levels of p65, p-IKKβ and p-IκBα increased significantly and the level of IκBα decreased distinctly in the abdominal aortic walls in the AAA group. However, zinc administration obviously reduced the levels of p65, p-IKKβ and p-IκBα, and prevented the IκBα degradation in AAA. In vitro experiment, TNF-α alone obviously increased the generation of p65, p-IKKβ and p-IκBα and reduced the degradation of the IκBα in the VSMCs compared with the control group, while the generation of the p65, p-IKKβ and p-IκBα clearly decreased and the IκBα significantly increased in the VSMCs in the presence of zinc. These results indicated that zinc could inhibit the canonical NF-κB pathway in AAA.

There are still some problems need to be solved. For the animals’ number limitation, the zinc deficient group was not involved at beginning of the experiment. Zinc deficiency can compromise the process in which zinc participate. The consequences of zinc deficiency are also diverse [46]. To further certificate the zinc’s effects in the process of the AAA, zinc deficient group should be included. In this study, we confirmed that zinc could induce A20 expression and inhibit the canonical NF-κB pathway in experimental rat’s AAA, but the effects of zinc on the non-canonical NF-κB pathway were not tested. In later experiment, the non-canonical NF-κB pathway will be investigated to understand the mechanisms about the effects of zinc on AAA. Additionally, there are still other mechanisms to suppress activation of IκB kinaseβ (IKKβ) and NF-κB, which cannot be ruled out from development and progression of the AAA. Furthermore, receptor interacting protein kinase 1(RIP1) is an essential member and only presents in the canonical NF-κB pathway. Results by Brian Skaug and Jueqi Chen et al showed that A20 was recruited to the IKKγ-RIP1 complex and inhibited IKK activation, apparently without deubiquitinating RIP1, causing degradation of RIP1 or disrupting the interaction of NEMO with polyubiquitinated RIP1 [47, 48]. But an opposite result showed A20 first cleaves lysine 63 (K63)-linked polyubiquitin chains on RIP1 and then conjugates lysine 48 (K48)-linked polyubiquitin chains that target RIP1 for degradation by the proteasome [49]. This contradictory may be caused by different cell types and experiment conditions.

In summary, the current study for the first time provide a convincing evidence that zinc can prevent the development and progression of CaCl_2_-induced AAA at least in part by induction of A20-mediated termination of the canonical NF-κB pathway. Zinc may be a potential therapeutic tool to development and progression of the AAA.

## Supporting Information

S1 Fig The morphology and identification of the primary culture VSMCs. (PDF)
