# Supplementary material for: Zinc Prevents Abdominal Aortic Aneurysm Formation by Induction of A20-Mediated Suppression of NF-κB Pathway
Source: PLoS One. 2016 Feb 26;11(2):e0148536. doi: 10.1371/journal.pone.0148536 (PMC4769024; doi:10.1371/journal.pone.0148536)
Supplement: S1 Fig — (PDF) [file pone.0148536.s001.pdf]

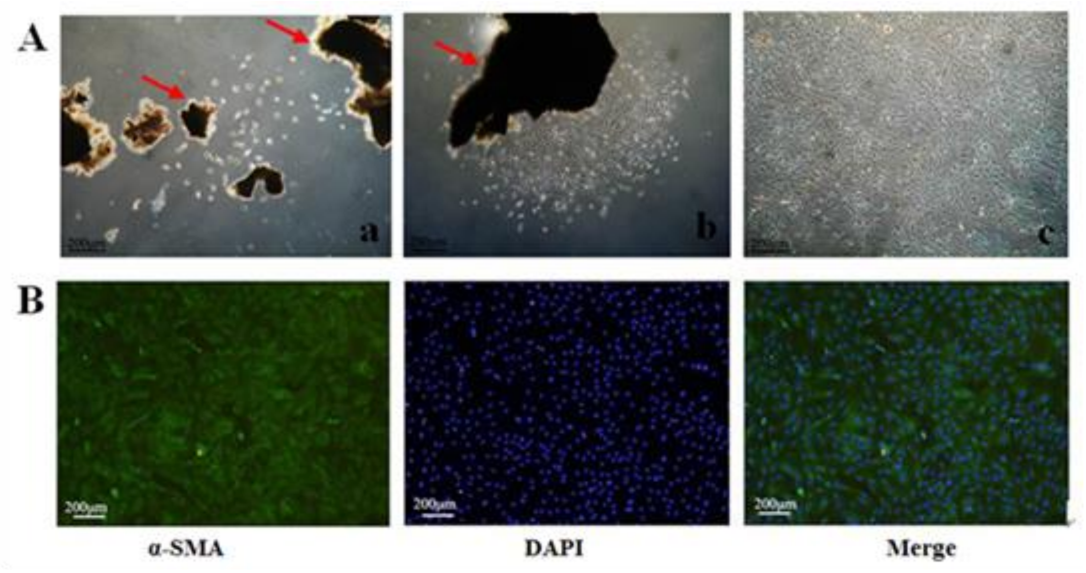

S1 Fig. The morphology and identification of the primary culture VSMCs. (A) The morphology of primary culture cells at 3 day (a), 7 day (b) and the 3rd passage (c). The red arrow points at tissue blocks of aortic wall. Bar = 200μm. (B) Cells were subjected to immunofluorescence staining with antibody against α-SMA (green) and nuclear staining with DAPI (blue), respectively. Bar = 200μm.
